# A thrips vector of tomato spotted wilt virus responds to tomato acylsugar chemical diversity with reduced oviposition and virus inoculation

**DOI:** 10.1038/s41598-019-53473-y

**Published:** 2019-11-20

**Authors:** Sulley Ben-Mahmoud, Taylor Anderson, Thomas M. Chappell, John R. Smeda, Martha A. Mutschler, George G. Kennedy, Darlene M. De Jong, Diane E. Ullman

**Affiliations:** 1 University of California, Department of Entomology and Nematology, Davis, CA 95616 United States of America; 2 Cornell University, Plant Breeding and Genetics Section, School of Integrative Plant Science, Ithaca, NY 14853 United States of America; 3 Texas A&M University, Department of Plant Pathology and Microbiology, College Station, TX 77843 United States of America; 4 University of Florida, Gulf Coast Research and Education Center, Wimauma, FL 33598 United States of America; 5 North Carolina State University, Department of Entomology and Plant Pathology, Raleigh, NC 27695 United States of America

**Keywords:** Behavioural ecology, Plant breeding

## Abstract

There is increasing evidence that acylsugars deter insect pests and plant virus vectors, including the western flower thrips (WFT), *Frankliniella occidentalis* (Pergande), vector of tomato spotted wilt virus (TSWV). Acylsugars are sugar-polyesters composed of saturated, un-saturated, and variously branched short and long chain fatty acids (FAs) esterified to a glucose (acylglucose) or sucrose (acylsucrose) moiety. We sought to understand how acylsucrose amount and composition of associated FA profiles interacted to mediate resistance to WFT oviposition and TSWV inoculation on tomato leaves. Towards this goal, we examined WFT oviposition and TSWV inoculation behavior on tomato lines bred to exude varying amounts of acylsucrose in association with diverse FA profiles. Our data show that as acylsucrose amounts increased, WFT egg-laying (oviposition) decreased and TSWV inoculation was suppressed. Western flower thrips also responded to FA profiles that included iC4, iC11, nC12 and nC10 FA. These findings support improving acylsugar-mediated resistance against WFT by breeding tomatoes exuding greater amounts of acylsucrose associated with specific FA profiles. We show that increasing acylsucrose amount output by type IV trichomes and selecting for particular FA profiles through advanced breeding profoundly affects WFT behavior in ways that benefit management of WFT as direct pests and as TSWV vectors.

## Introduction

The western flower thrips (WFT), *Frankliniella occidentalis* (Pergande), is an important pest of numerous crops including tomato, *Solanum lycopersicum* (L.) (hereafter “tomato”). Western flower thrips cause direct damage to plants by rupturing leaf, flower, and fruit cells when feeding, leaving behind silvery patches and fruit lesions that reduce plant yields and tomato marketability^1,2^. Adult females also cause damage when they puncture plant tissue to embed their eggs (oviposition). Western flower thrips cause indirect damage to plants by transmitting tomato spotted wilt virus (TSWV), resulting in massive crop losses worldwide^3,4^. After hatching from eggs on TSWV-infected plants, larval WFT acquire TSWV. Acquisition at the larval stage is a prerequisite for TSWV passage between WFT developmental stages, and eventual infection of the principal salivary gland of the adult, after which the insect may inoculate new hosts^5–8^. Strategies to reduce WFT oviposition can prevent population increases thus limiting direct damage to crops, as well as enhancing management of TSWV by reducing production of infective adults that inoculate new host plants and cause secondary spread of the virus.

Currently, growers attempt to manage WFT and TSWV spread by reducing insect populations with insecticides, and/or by planting tomato cultivars with the *Sw-5* gene that mediates TSWV resistance^9–11^. Chemical insecticides can be harmful to the environment, humans, and non-target organisms^12^ and pesticide resistance is common among WFT populations^13,14^. In addition, *Sw-5* resistance-breaking isolates are increasingly detected^9–11,15,16^. Producers need novel strategies, like the deployment of acylsugar-mediated thrips resistance, to improve management of WFT, limit spread of TSWV in tomato crops, and advance the goals of safe and sustainable agricultural production^7,17–19^.

Specialized glandular trichomes on aerial portions of tomato plants produce and exude secondary metabolites called acylsugars^20–22^, which cause insect-repellent and antibiosis effects that affect behavior of several species of important tomato pests, including WFT^20–29^. These complex molecules are sugar-polyesters composed of saturated, un-saturated, and variously branched short and long chain FAs, esterified to a glucose (acylglucose) or sucrose (acylsucrose) moiety^30–32^. Great diversity occurs in the FAs and we know little about the functions of their individual components. Thus, we describe the relative amount and type of FAs in a plant’s acylsugar exudate as a FA profile. The sugar moiety, acylsugar amount and/or FA profile can vary considerably, as shown by the great diversity of acylsugars produced by wild relatives of tomato^33–35^.

The wild tomato relative, *Solanum pennellii* Correll, is rich in type IV glandular trichomes that exude acylsugar droplets, whereas cultivated tomato produces very little acylsugar even though trichomes similar to type IV glandular trichomes are present in varying densities^31,32^. Thus, tomato breeding efforts have concentrated on transferring the quantitative trait loci (QTL) that underlie increased acylsugar production or types of acylsugars produced from *S. pennellii* (Correll) D’Arcy accession LA716 to tomato^29,36–38^. Breeding by the Cornell tomato-breeding program led to creation of acylsugar benchmark line, CU071026^26^. This line accumulates approximately 15% of the extremely high acylsugar amount produced by its *S. pennellii* LA716 parent. The acylsugars of CU071026, are sufficient to suppress silverleaf whitefly, *Bemisia tabaci* (Gennadius), oviposition on plants in field cages and WFT oviposition in sepals^26,29^.

Additional acylsugar QTL affecting density of type IV trichomes, amount of acylsugar exudate, sugar moiety, and FA profile were identified^27,39–41^ and introgressed into CU071026 via marker-assisted backcrossing^28,37,42^, creating a series of tomato lines that we call “acylsugar lines”, that differ in density of type IV trichomes, amounts of exuded acylsugars, predominant sugar moieties, and FA profiles. The impact predicted for each of the QTL transferred was confirmed by the impacts that QTL had on the acylsugars produced by the resulting new acylsugar line^26,28,37,42^. These acylsugar lines form a research platform for *in planta* trials of the impact of differences of acylsugar amount, sugar moiety, and/or FA profile on insect oviposition, longevity or insect inoculation of viruses. Results from previous studies show that variation for acylsugar amount and composition is biologically relevant to insect interactions^26,29,36,37^.

Evaluation of acylsugar lines in field, greenhouse and laboratory settings gave additional evidence supporting the utility of endogenously produced or exogenously applied acylsugars in thrips management^29,37^ (Chappell *et al*., unpublished). A study focusing on flowers of tomato hybrids and acylsugar lines involving diverse combinations of acylsucrose and acylglucose moieties and multiple FA profiles showed that increasing total amount of acylsugar resulted in reduced WFT oviposition. Also, the presence of particular FAs (iC5, iC9 and iC11) in FA profiles was associated with variation in WFT oviposition on flowers, suggesting that FA profile, in conjunction with acylsugar amount, plays a role in mediating WFT behavior, and this relationship warranted more in-depth study^29^. The study could not tease apart the specific effects of different sugar moieties, nor did it address TSWV inoculation or WFT responses to leaves. Understanding WFT responses to leaves is important because insects often colonize foliar parts of the plant and infection of young plants with TSWV prior to flowering frequently results in severe agricultural losses^3^. Investigation of foliar acylsugar effects on WFT showed that when trichomes exuding acylglucose as the predominant sugar moiety were combined with particular FA profiles, WFT oviposition and inoculation of TSWV were effectively suppressed as amount of acylglucose increased^37^. Based on these collective findings, we wanted to understand whether variation in acylsucrose amount and its interaction with specific FA profiles would also affect WFT oviposition and inoculation of TSWV. To explore this question, we measured WFT oviposition responses and TSWV inoculation efficiency on acylsugar lines bred to exude varying amounts of acylsucrose and diverse FA profiles^26,28,37,42^. Our data from choice tests that compared WFT responses to leaf discs with exuded acylsugars (unwashed) and leaf discs that we washed to remove acylsugar exudates, show for the first time that increased acylsucrose amount and FA profiles interact to mediate resistance to WFT oviposition and TSWV inoculation on leaves.

## Results

### Chemical analysis of acylsugar exudates

We characterize acylsugar exudate from the leaf of a tomato or acylsugar line by the total amount (µmol g^−1^ dry leaf weight) of acylsugar exuded, the proportion of these acylsugars with a glucose/sucrose sugar moiety, and the types and relative amounts of FAs attached to the sugar moiety. A full characterization of an acylsugar lines’ FA profile also includes identification of the attachment point(s) of FA chains on the sugar ring. The acylsugar exudates of lines used in this study have been previously characterized^26,28,37,42^. We analyzed exudates for the specific plants used in our experiments to control for the possibility that acylsugar chemical composition could vary with growing conditions and because we were interested in understanding WFT responses to varying acylsucrose amounts and FA profiles.

**Figure 1:**
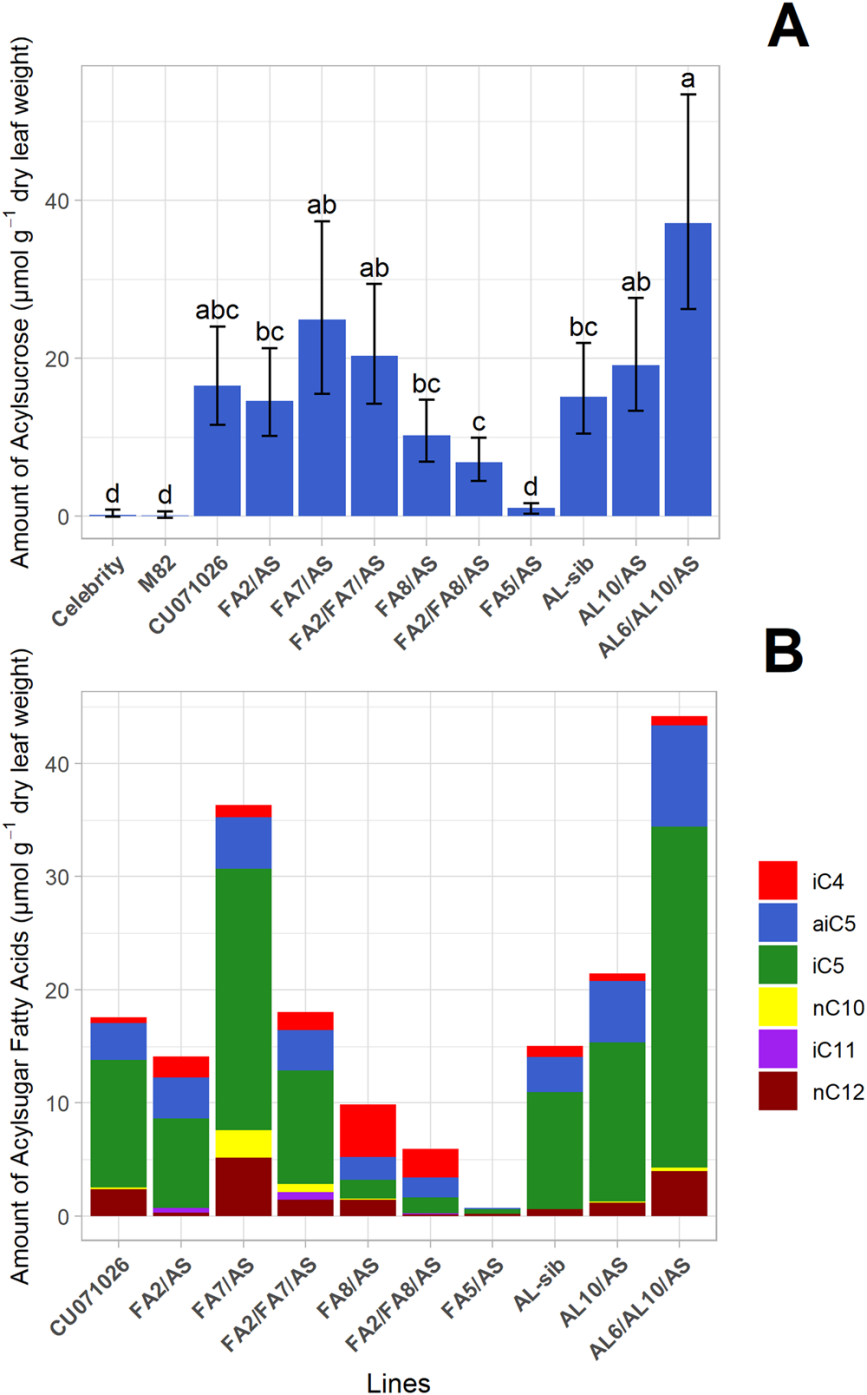
Average amounts of foliar acylsucroses (**A**) and average amounts of acylsugar fatty acids (FAs) (**B**) for the acylsugar lines and tomato controls. Leaves were sampled from 12-week old plants (4 leaflets per plant, 3 plants per line; leaves were identical to those sampled for oviposition and inoculation experiments). The average amounts of accumulated acylsucroses (±95% confidence intervals) are measured in µmol g^−1^ of dry leaf weight. (**A**) There were significant differences in acylsucrose accumulations among acylsugar lines and tomato controls. Lines with the same letter are not significantly different by Tukey HSD (α = 0.05). The non-acylsugar controls, Celebrity and M82, accumulated negligible acylsugar relative to CU071026 and to derived acylsugar lines containing *S. pennellii* LA716 QTL that either increased a line’s acylsucrose amount (Line name suffix = “AS”) or the FA profile of a modified line’s acylsucroses (Line name prefix = “FA”). (**B**) The predominant FAs (FAs that constituted ≥5%, by weight, of the total FA profile of at least one line) measured in each acylsugar line’s leaf acylsucroses are shown. The amounts of acylsugars, and their associated FAs, for Celebrity and M82 were below the limit of quantitation and are not shown.

The commercial tomato cultivars, Celebrity and M82, accumulated only trace amounts of acylsugar, while CU071026 and most of its derived acylsugar lines accumulated much higher amounts of acylsugar, specifically acylsucrose (Fig. 1A). The amounts of foliar acylsucrose varied significantly (F_11,59_ = 24.459, P < 0.001) across all entries (tomato control and acylsugar lines), and among the acylsugar lines (F_9,49_ = 14.876, P < 0.001) (Fig. 1A). No acylsugar line accumulated more than 1.2% acylglucose by weight, revealing acylsucrose to be the dominant sugar moiety for all the lines. Consistent with the findings of Smeda *et al*.^42^, the acylsugar line FA5/AS produced only trace amounts of acylsugar as compared to the acylsugar benchmark line CU071026. FA5/AS possesses all of the LA716 introgressions in CU071026, and the trichomes that would produce acylsugars are present at similar density as CU017026; however, the additional chromosome 5 introgression in FA5/AS contains an acylhydrolase-encoding QTL that disrupts acylsugar accumulation. Hence, FA5/AS serves as a control for the effects of foliar trichomes in the absence of acylsugar exudates on WFT behavior. Under our experimental conditions, AL10, a breeding line that contains the chromosome 10 QTL introgression (QTL10) that impacts acylsugar amount, did not accumulate more acylsugar than its sibling isoline, AL-sib that lacks the chromosome 6 QTL (QTL6) and chromosome 10 QTL introgressions impacting acylsugar amount (Fig. 1A). However, AL6/AL10/AS, which has both QTL6 and QTL10, accumulates approximately 2-fold the acylsugar amount of AL-sib, which is consistent with prior tests of these acylsugar lines^26^.

Chemical characterizations also showed that breeding lines that have additional introgressions that contain FA profile modifying QTL (lines FA2/AS, FA7/AS, FA2/FA7/AS, FA8/AS, and FA2/FA8/AS) accumulated varied amounts of acylsucrose (Fig. 1A), indicating possible pleiotropy of the underlying QTL on both acylsucrose amount and acylsugar FA profile. These findings are also consistent with results of Smeda *et al*.^37,42^, which detailed the creation and characterization of each of these acylsugar lines.

Chemical analysis of the acylsugar exudates by GC-MS showed the acylsugar lines to have different FA profiles (Figs. 1 and S1). The total amounts (µmol g^−1^ dry leaf weight) of the predominant FAs (those that constitute ≥5% of the overall FA profile of at least one acylsugar line) for each line are shown in Fig. 1B. While we have described the FA profiles of these lines previously in detail^22,27,28,30,37^, we confirm herein the FA profiles of the specific plants used in our experiments. The predominant FAs in this experiment included the iso-branched 4, 5 or 11-carbon acyl group FAs iC4, iC5 and iC11, respectively, an anteiso-branched 5-carbon acyl group FA aiC5, and the straight chain 10 or 12-carbon acyl FAs nC10 and nC12. A hierarchical clustering analysis (Fig. S1) done to investigate the relative “distance” between acylsugar lines (calculated from their FA profiles), indicated that lines with QTL modifying acylsucrose amount have FA profiles that are very similar to each other and to the benchmark line CU071026. However, lines with QTL that modify the FA profiles are clustered progressively further from CU071026, indicating dissimilarity from this acylsugar benchmark line. There were no high-level divisions among the acylsugar lines in the clustering analysis, precluding investigations into how high-level changes in clustered FA profiles might affect oviposition or TSWV inoculation rates. The QTL with the greatest individual impacts on FA profiles were FA2 and FA7 (Fig. S1). Interestingly, in the line FA2/FA8/AS, which combines QTL from FA2/AS and FA8/AS, we observed a FA profile that was different from the benchmark line (CU071026) due to notable increases in production of the precursor species iC4 and in longer-chained FAs, such as iC10 and iC12 (Fig. S1). We also found that production of many FAs were positively or negatively correlated across the lines, as depicted by the column clustering in Fig. S1, and the FA correlation heatmap in Fig. S2. The results in the following section show how differences in FA profile affect the probability of WFT oviposition and TSWV inoculation.

### Removal of leaf acylsugars for bioassays

We tested washing of leaf discs with various liquids to optimize removal of foliar acylsugars while avoiding physical damage to leaf discs and trichomes. Liquids tested included water, tween, ethanol, and hexane. We applied these liquids with a firm spray from a wide-bore squirt bottle. Microscopic examination of the leaf discs after each wash showed that while ethanol was the most effective solvent for removing acylsugar droplets, it damaged the cuticle, potentially confounding thrips choices. While water does not dissolve acylsugars, which are non-polar, water efficiently removed acylsugar droplets, without damaging the trichomes or cuticle. Furthermore, water was unlikely to leave behind residues that could influence thrips choices, unlike the other solvents tested. Examination of water washing efficiency using CU071026 leaf discs (Supplementary Data 1) revealed a strong initial reduction (~77%) of acylsugars on washed leaf discs immediately after washing compared to unwashed leaf discs (*t*_28_ = 8.93 *p* < 0.001). Based on these data, we used water to wash leaf discs in all the oviposition and TSWV inoculation choice experiments.

### Western flower thrips oviposition onto leaf discs from acylsugar lines and tomato controls

**Figure 2:**
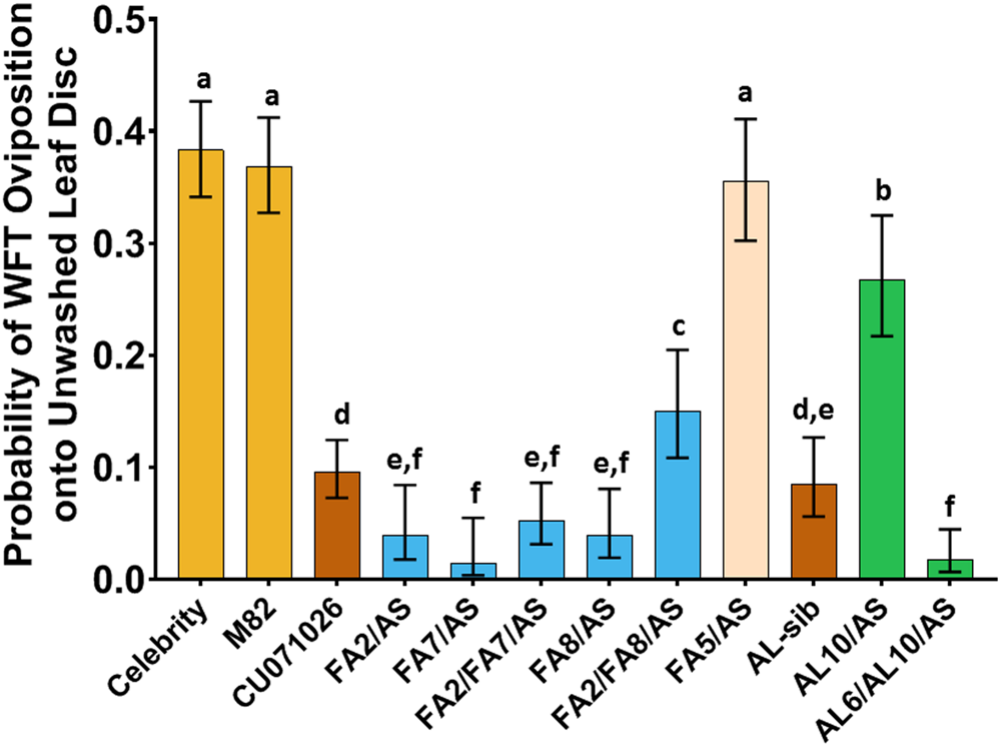
Reduced western flower thrips (WFT) oviposition onto acylsugar lines relative to non-acylsugar controls. Oviposition arenas contained one washed and one unwashed leaf disc of an acylsugar line or control onto which a cohort of 10 female non-infected WFT were released to oviposit. Eggs laid onto leaf discs were counted to estimate their probability of oviposition which was analyzed as a binary response. Mean probability of oviposition (±95% Confidence limits) onto unwashed leaf discs of respective acylsugar lines is denoted by colored bars. Acylsugar lines in the same category are indicated by uniform colors: Celebrity and M82 (yellow bars) are non-acylsugar control lines; CU071026 (acylsugar benchmark line) and AL-sib (brown bars; acylsugar amount sibling line lacking QTL6 or QTL10) are acylsugar control lines. FA5/AS shares all but one introgression with CU071026, but has negligible acylsugar production due to the FA5 QTL. Fatty acid (FA) lines (blue bars) differ by modifications of FA and additionally in some cases by acylsugar amount, and AL acylsugar lines (green bars) exude increased amounts of acylsugars and when QTL6 is present, possess increased type IV trichome density. Acylsugars significantly decreased the probability of WFT oviposition onto unwashed leaf discs. Comparison of oviposition onto unwashed leaf discs across acylsugar lines reveals that, in general, the lines exuding moderate to high amounts of acylsugar significantly deterred WFT from laying eggs onto unwashed leaf discs, relative to the tomato controls and to the non-acylsugar producing fatty acid line FA5/AS. Lines with the same letter are not significantly different by Tukey HSD (α = 0.05).

Choice experiments offered WFT pairs of leaf discs that differed in the presence or absence of acylsugar (unwashed or washed leaf discs, respectively) as substrates for oviposition. The probability of oviposition on unwashed leaf discs of the acylsugar lines was significantly lower than on the tomato controls, Celebrity and M82, and the trichome density control line FA5/AS (Fig. 2). These data confirm that acylsucroses, and not the type IV trichomes or other gene products encoded by *S. pennellii* introgressions, underlie deterrence of WFT oviposition. In general, as acylsucrose amount increased, the probability of WFT oviposition decreased (Fig. S3). For example, among the subset of lines with QTL increasing either type IV trichome density and/or acylsucrose amount (AL10/AS and AL6/AL10/AS, Fig. 2), AL6/AL10/AS produced the greatest amount of acylsucrose relative to any other line (Fig. 1A) and significantly reduced the probability of oviposition relative to controls and many other acylsugar lines. However, acylsucrose amount did not always explain suppression of WFT oviposition. For example, the probability of oviposition on AL10/AS (a line bred for increased type IV trichome density) was significantly higher than other acylsugar lines and the sibling isoline AL-sib, which lacks QTL6 and 10. In addition, despite wide variation for acylsucrose amount among lines with altered FA profiles (blue bars in Fig. 2), the probability of WFT oviposition was similarly suppressed among these lines, with the exception of FA2/FA8/AS. As noted in our chemical analysis, FA2/FA8/AS has a different FA profile than the other FA lines and WFT oviposited significantly more eggs on FA2/FA8/AS than the other FA lines (blue bars in Fig. 2). These findings suggest that characteristic(s) beyond total acylsucrose amount, in particular FA profiles, play important roles in reducing the probability of WFT oviposition.

### Western flower thrips TSWV inoculation onto leaf discs from acylsugar lines and tomato controls

**Figure 3:**
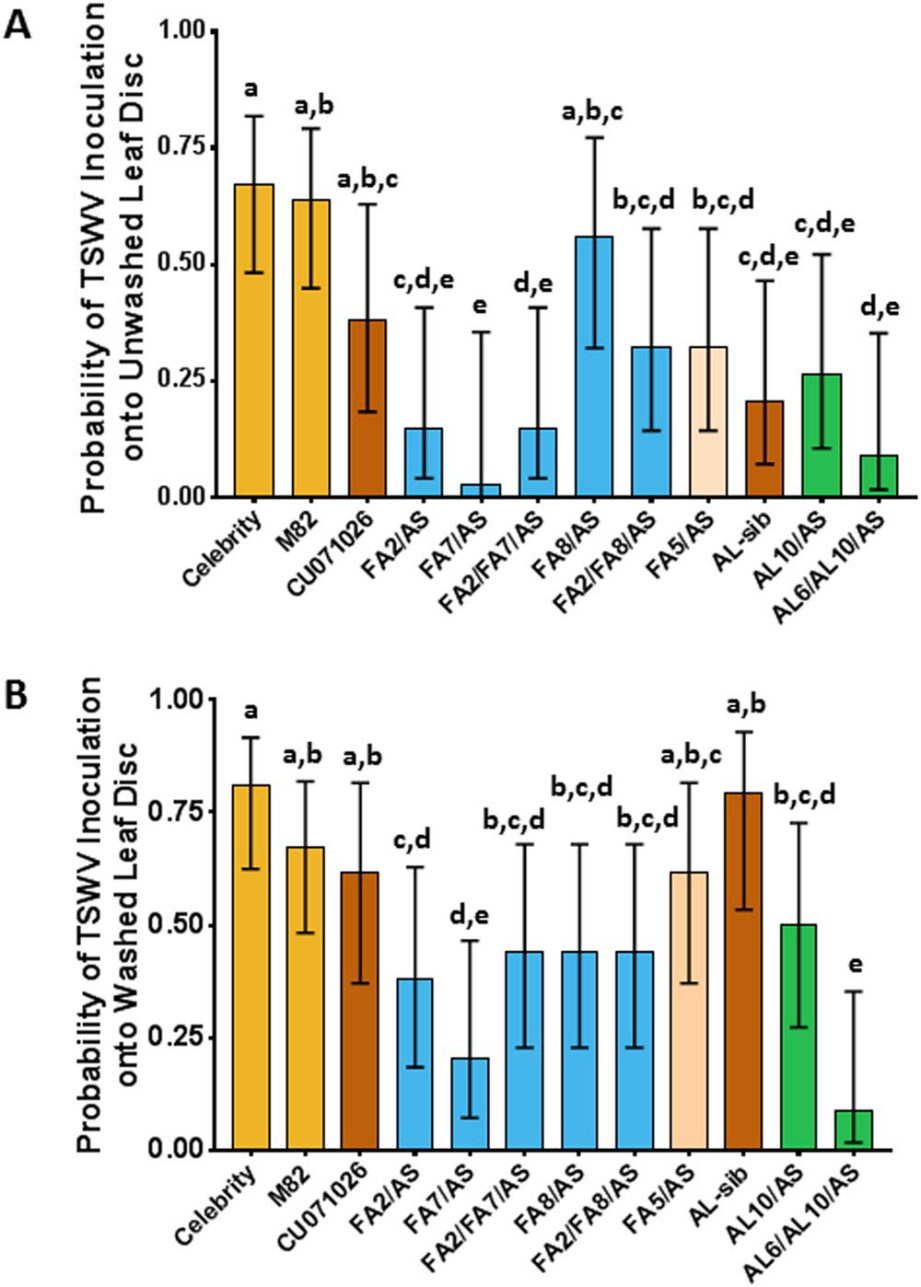
Reduced tomato spotted wilt virus (TSWV) inoculation by western flower thrips (WFT) onto acylsugar lines relative to non-acylsugar controls. Inoculation arenas contained one unwashed (probability of TSWV inoculation shown in (**A**) and one washed leaf disc (probability of TSWV inoculation shown in (**B**) of a tomato or acylsugar line into which four WFT from a TSWV-infected colony were released to inoculate virus. TSWV-infected leaf discs were detected by ELISA to estimate the mean probability of TSWV inoculation (±95% confidence limits) onto respective leaf discs of tomato lines. Acylsugar lines or controls in the same category are indicated by uniform colors: Celebrity and M82 are non-acylsugar tomato controls; CU071026 (acylsugar benchmark line) and AL-sib (acylsugar amount sibling line lacking QTL6 or QTL10) are acylsugar amount controls, fatty acid (FA) lines (in blue) differ by modifications of FAs, except for the FA5/AS control line which has negligible acylsugar production due to the impact of QTL5. The AL acylsugar lines (in green) exude increased amounts of acylsucroses without altering acylsucrose FA profile or sugar moiety, and possess increased type IV trichome number in the presence of QTL6. Probability of TSWV inoculation onto washed/unwashed leaf discs was compared across acylsugar lines and tomato controls. Generally, the acylsugar line leaf discs reduced the probability of TSWV inoculation onto their unwashed/washed leaf discs, compared to leaf discs of the tomato controls and the negative acylsugar control - FA5/AS, and their ability to deter TSWV inoculation was reduced when acylsucroses were washed away. Lines with the same letter are not significantly different by Tukey HSD (α = 0.05) for respective unwashed/washed leaf discs.

The probability of TSWV inoculation by WFT was highest on the Celebrity and M82 controls regardless of washing (Fig. 3A,B). In contrast, acylsucrose exuding lines FA2/AS, FA7/AS, FA2/FA7/AS, AL-sib, AL10/AS and AL6/AL10/AS significantly decreased the probability of TSWV inoculation by WFT relative to that of the Celebrity and M82 controls, the acylsugar benchmark, CU071026, and FA lines FA8/AS, FA2/FA8/AS and FA5/AS (Fig. 3A). The greatest reduction in the probability of TSWV inoculation was on FA7/AS, although it was not significantly different from that on FA2/FA7/AS, AL-sib, AL10/AS and AL6/AL10/AS. Among the acylsugar lines, removal of acylsugars with washing resulted in a greater probability of TSWV inoculation by WFT, with the exception of AL6/AL10/AS (Fig. 3B). Increasing acylsucrose amount resulted in decreased probability of TSWV inoculation (Fig. S4); however, acylsucrose amount alone did not fully explain suppression of TSWV inoculation by WFT. For example, the benchmark acylsugar line CU071026 did not significantly deter WFT inoculation of TSWV relative to the Celebrity and M82 controls that produce only trace acylsugars (Fig. 3A). Additionally, CU071026 produced acylsucrose at a similar amount, but with a different FA profile (Fig. 1) as lines that were more effective at suppressing TSWV inoculation, such as FA2/AS and FA7/AS (Fig. 3A).

### Identification of acylsugar chemical characteristics predictive of western flower thrips oviposition and TSWV inoculation

To better understand the impact of foliar acylsugars on WFT behavior, we conducted choice experiments in which WFT oviposition was interpreted based on a choice between two leaf discs of the same acylsugar line or control, one exuding acylsugars and one that had its acylsugars washed away. When we gave insects this choice, the probability of WFT ovipositing on an unwashed leaf disc was negatively correlated with the average amount of foliar acylsucrose for that line (Fig. S3). For each increase of one µmol acylsugar per gram of dry leaf weight (µmol g^−1^), the odds of oviposition on the unwashed leaf disc relative to the washed leaf disc decreased by a factor of 0.910 ± 0.011. We investigated TSWV inoculation similarly, though not as a binary choice. When given a choice between two leaf discs of the same acylsugar line or control, one exuding acylsugars and one that had its acylsugars washed away, the odds of virus inoculation in the unwashed leaf disc decreased by a factor of 0.918 ± 0.030 for an average increase of one µmol acylsugar g^−1^ of dry leaf weight (Fig. S4). The odds of virus inoculation on washed leaf discs also decreased by a factor of 0.936 ± 0.026 for each increase of one µmol acylsugar g^−1^ of dry leaf weight. While the probability of TWSV inoculation onto both the washed and unwashed leaf discs diminished with respect to acylsugar amount, inoculation rates for washed leaf discs were significantly higher for a large range of acylsugar amounts (Fig. S4).

Because the relationship between total acylsugar exudate and WFT behaviors varied depending on the composition of the exudate, we tested the hypothesis that FA profile may cause variation in per-unit effect of different exudate components. We investigated this hypothetical effect by analyzing the amounts of FAs measured for each line as potential independent variables. Best-fit models were chosen by minimization of Akaike Information Criterion (AIC), and multicollinearity between independent variables was addressed as follows. The optimized oviposition model revealed that amounts of three FAs: iso-branched four-carbon FA (iC4), iso-branched eleven-carbon FA (iC11), and straight-chain twelve-carbon FA (nC12), explained significant variation for WFT oviposition choices after accounting for the variation due to acylsucrose amount (Table 1). Among these FAs, amount of iC11 per unit, had a greater effect on suppression of WFT oviposition than did iC4 and nC12, though iC4 and nC12 were more prevalent in the exudates from the acylsugar lines we tested (Fig. 1B). The best TSWV inoculation model included the straight-chain ten-carbon FA (nC10) and total acylsucrose amount (Table 2). Increases in nC10 were associated with reductions in the probability of TSWV inoculation by WFT.

**Table 1:** Regression model showing acylsugar fatty acids associated with reductions in *Frankliniella occidentalis* oviposition onto leaf discs.

	Analysis of Deviance (Type III Tests)				
	Estimate	Std. Error	Likelihood χ^2^	df	p-value
Intercept^a^	−0.485	0.119	—	—	—
Acylsugar amount	−0.019	0.013	2.04	1	p = 0.153
iC4	−0.385	0.069	42.35	1	p < 0.001
iC11	−1.376	0.440	11.19	1	p = 0.001
nC12	−0.501	0.102	26.00	1	p < 0.001

Reduction in the probability of oviposition onto leaf discs of acylsugar lines can be explained by acylsucrose amount and the types of acylsugar fatty acids accumulated on the leaves. Model coefficients were estimated by Firth’s bias-reduced logistic regression. Model fit statistics following the inclusion of parameters are indicated by the Type III analysis of deviance.

^a^Intercept indicates the log odds of oviposition into an unwashed leaf disc in the absence of acylsugars and their fatty acids.

**Table 2:** Regression model showing acylsugar fatty acids associated with reductions in *Frankliniella occidentalis* TSWV inoculation onto leaf discs.

	Analysis of Deviance (Type III Tests)				
	Estimate	Std. Error	Likelihood χ^2^	df	p-value
Intercept^a^	−0.314	0.326	—	—	—
Acylsugar amount	−0.032	0.022	1.76	1	0.184
nC10	−1.256	0.663	7.16	1	0.007

Reduction in the probability of tomato spotted wilt virus inoculation onto leaf discs of acylsugar lines can be explained by total acylsugar amount, and the type of acylsugar fatty acids accumulated on their leaves. Model coefficients were estimated by Firth’s bias-reduced logistic regression. Model fit statistics following the inclusion of parameters are indicated by the Type III analysis of deviance.

^a^Intercept indicates the log odds of oviposition into an unwashed leaf disc in the absence of acylsugars and their fatty acids.

While we cannot separate pairs or groups of collinear independent variables (Fig. S1) by associative methods with our current data, there is a biological basis for understanding why these variables are correlated. Previous investigations have shown how FAs are associated in biochemical pathways^39^. For example, abundance of nC10 and nC12 are correlated because nC10 is a precursor of nC12 in acylsugar biosynthesis. Accordingly, these results do not address the relative importance of nC10 vs nC12 in affecting WFT behavior, but do indicate that one or both of these FAs are relevant. The same effect applies to iC9 and iC11, where iC9 is the immediate precursor to iC11. Future breeding efforts that are able to manipulate amounts of these FAs independently will clarify their relative importance.

## Discussion

Our data show that increased acylsucrose amount and specific acylsugar FA profiles interact to mediate resistance to WFT oviposition and TSWV inoculation on leaves. These findings advance the prospects of managing WFT with acylsugar-mediated resistance by identifying how specific acylsugar components (sugar moiety, amount, FA profile) contribute to suppression of WFT oviposition and TSWV inoculation on leaves. Support for the importance of our findings is found in previous research showing the efficacy of diverse acylsugars against multiple insect pests with *in vitro* assays using purified acylsugars^20,21,24,25,36^, and with *in planta* assays showing the role of total acylsugar amount and trichome density on insect control^26,29,37^. The role of diverse acylsugars and FA profiles in suppressing WFT oviposition on tomato flowers has been shown^29^; however, none of these investigations explored the contributions of different acylsugar moieties on WFT control, or the effect of FA profiles on WFT oviposition or inoculation of TSWV on leaves. Research aimed at the impact of acylglucoses and associated FA profiles showed that acylsugar lines that produced acylglucoses at or above the amount of acylsucrose accumulated by the benchmark line CU071026 along with specific FA profiles significantly suppressed WFT oviposition and TSWV inoculation^37^.

The current investigation used acylsugar lines bred to exude predominantly acylsucroses, and focused specifically on the role of acylsucrose amount, and associated FA profiles on suppression of WFT oviposition and TSWV inoculation. Our results show that increased amounts of acylsucrose significantly suppressed WFT oviposition and TSWV inoculation (Figs. S3 and S4). It was also evident that amount of acylsucrose did not fully explain the changes we observed in WFT behavior. Our analysis also shows that particular FA profiles containing specific FAs also affected suppression of WFT oviposition and TSWV inoculation. Together, the results of this study and multiple previous investigations^26–29,36,37,42^, support the conclusion that type of sugar moiety is less important in the suppression of WFT oviposition and TSWV inoculation than having an adequate amount of acylsugar exuded. Specifically, as amount of acylsugar increases, WFT oviposition and TSWV inoculation is increasingly suppressed. Furthermore, our results show that the FA profile of the acylsugars exuded by trichomes on leaves is also an important determinant of WFT behavior. This finding supports prior results investigating WFT oviposition on flowers^29^. Thus, adequate amounts of either acylsucroses or acylglucoses are likely effective in suppressing WFT oviposition and TSWV inoculation when combined with an effective acylsugar FA profile. The precise elements of the acylsugar FA profiles that modulate WFT responses need more study, including, but not limited to, which FAs are most significant, the role of FA attachment to the sugar moiety and the branching pattern of the acylsugars FA.

Management of TSWV spread by WFT is a serious problem worldwide and our investigations show that particular combinations of acylsucroses (this study) or acylglucoses^37^ with specific FA profiles can deter WFT inoculation of TSWV. These findings advance the prospects of managing WFT by exploiting natural tomato resistance as part of integrated pest management strategies. The potential that acylsugar lines could protect against multiple insect pests and the viruses they transmit is important as the international community works towards safer and more sustainable methods of pest management. This concept is supported by past evidence showing acylsucroses deterred settling and probing behaviors by the whitefly *Bemisia tabaci* (Genn.), and reduced the transmission of *Tomato yellow leaf curl virus* (TYLCV)^43,44^. Finally, our findings demonstrate the utility of the acylsugar lines as a research tool, and provide a significant step towards understanding the importance of FA profiles and specific acylsugar moieties in mediating WFT control, opening the door to in-depth analysis of their role in suppressing multiple tomato pests.

## Methods

### Organisms

Western flower thrips colonies (non-infected and TSWV-infected) were maintained using protocols described previously^45^. We used adult females, 2–6 days post-pupal-eclosion, for oviposition and TSWV inoculation choice experiments. To infect WFT with TSWV, we acquired first instar larvae from a non-infected colony a few hours after hatching, then provided them with TSWV-infected *Datura stramonium* L. leaves for 12–16 hours, after which we maintained the thrips on green bean pods, *Phaseolus vulgaris* L.

Acylsugar lines and tomato controls used in this study are described in detail elsewhere^26,28,37,42^. Briefly, the commercial tomato cultivars, Celebrity and M82, were included as “trace” or non-acylsugar controls. The Cornell benchmark acylsugar line, CU071026, was included as an acylsugar positive control for consistent comparison with previous studies^26–29,36,37,42^. Eight acylsugar lines (suffix “AS”) that share CU071026 as the recurrent parent were used: FA2/AS, FA5/AS, FA7/AS, FA8/AS, FA2/FA7/AS, FA2/FA8/AS, AL10/AS and AL6/AL10/AS^42^. Fatty acid lines (prefix “FA”) possess QTL known to modify their acylsugar FA profile^27^: FA2/AS^42^, FA5/AS^42^, FA7/AS^42^, FA8/AS^42^, FA2/FA7/AS^28^ and FA2/FA8/AS^28^. Line FA5/AS has trichome densities similar to the other FA lines, but exudes acylsugars at very low amounts^37^ and is therefore included to control for the possibility that type IV trichome presence alone impacts insect responses differentially. Lines bred for increased trichome number, and/or acylsugar production have the prefix “AL” and include AL-sib, AL10/AS and AL6/AL10/AS. AL-sib^26^ (Mutschler *et al*. unpublished) was obtained from the same BC_1_F_2_ population that generated the increased acylsugar production lines but was selected to recover the CU071026 genotype, and is therefore a closely related control line.

Seeds of the above acylsugar lines and tomato controls were grown in a greenhouse at the University of California, Davis (UCD). Seedlings were transplanted individually to 1-gallon pots (McConkey, Sumner, Washington) six weeks after germination, and placed randomly and equidistantly on benches. Drip irrigation delivered nutrients to plants to avoid compromising trichomes or acylsugar exudates. We trimmed plants periodically to prevent contact between leaves and between plants. Paired leaflets adjacent to the terminal leaflet on the first fully expanded leaf were collected for acylsugar chemical analysis, and for use in WFT oviposition and TSWV inoculation leaf disc assays. To ensure acylsugars and trichomes were undisturbed during transit for bioassays, we placed leaflets adaxial side down, in an enclosed plastic humid-box lined with moist paper towels.

### Chemical analysis of acylsugar exudates

Acylsugar exudate accumulated by acylsugar lines and tomato controls was quantified when plants were 12-weeks old using previously described methods^27^. We sampled two sets of paired leaflets from three plants per line to determine their chemical characteristics. Each leaflet pair was placed in a 20 ml HDPE scintillation vial (Fisherbrand^TM^) and air-dried. Dried leaf samples were rinsed with 3 ml of methanol, and the rinsate was used for downstream analysis as described by Leckie *et al*.^26,27^. The amounts of acylsugar FAs were measured as previously described^27,28,42^.

### Removal of leaf acylsugars for bioassays

To find an efficient technique to remove acylsugars from leaf discs for bioassays, trials were conducted with water, Tween (0.5–2%), ethanol (70–95%) and hexane. Leaf discs of CU071026 were cut with a 16 mm cork-borer and washed under a steady stream of the aforementioned solvents for 10 sec, using a customized wide-bore Nalgene^TM^ squirt bottle to wash away foliar acylsugar-exudate, followed by a spectrophotometric assay (see chemical analysis of leaves) to assess acylsugar removal. Based on these results, we selected water as the solvent of choice for all subsequent experiments.

### Western flower thrips oviposition into leaf discs

Leaf discs (16 mm diameter) were set up in six independent oviposition choice experiments, made up of five replicates of each acylsugar line and tomato controls. For each of these replicates, ten non-infected gravid female thrips (2–6 days post-eclosion adults) were offered a pair of leaf discs: ‘washed’ (acylsugars removed) and ‘unwashed’ leaf disc (acylsugars intact), in arenas (Pall petri dishes, height × diameter: 9.0 × 50.0 mm) with lids modified with screens. Leaf discs were washed and dried briefly, prior to placement in oviposition arenas. Chilled, female WFT were placed mid-way between the pair of leaf discs with a fine brush. Leaf-discs were embedded, abaxial-side-up, atop 1 mm thick 1% agar discs to prevent desiccation. To check for the possibility that WFT oviposition responses could be influenced by factors other than acylsugars, additional controls were set-up alongside choice-experiments that included arenas of two unwashed leaf discs of ‘Celebrity’ (n = 20). Oviposition arenas were randomly placed under a constant fluorescent light source at room temperature for 24 hr prior to staining and counting of eggs, using a method previously described for staining leafhopper eggs^46^. Western flower thrips eggs were visualized and counted under a light microscope (Leica MZ125) following staining with McBrides solution (5 ml, 0.2% acid fuchsin in a 1:1 solution composed of 95% ethanol and glacial acetic acid) and de-staining with lactic acid:glycerol:water (1:1:1). Staining of leaf discs was conducted at room temperature (8 hr) whilst shaking at low speed (model 4625, Lab-line instruments, Melrose Park, IL). De-staining was done in an incubator at 80 °C for at least 72 hr, interspersed with 30 min shaking at a low speed every 24 hr.

### Western flower thrips TSWV inoculation onto leaf discs

Pairs of washed versus unwashed leaf discs of acylsugar lines or tomato controls were prepared and staged in arenas as described previously. Four adult females (24–72 hr post eclosion), from the TSWV-infected thrips colony, were added to each arena for a 24 hr inoculation access period (IAP). Virus inoculation arenas were randomized under a fluorescent light source at room temperature. After the IAP, thrips were removed, and leaf discs incubated at room temperature for 48 hr, after which TSWV infection status of WFT and leaf discs was determined by enzyme-linked immunosorbent assay (ELISA) reagent set for TSWV (Agdia, Elkhart, IN). Five experimental replicates of each acylsugar line or control constituted an inoculation experiment, and we performed five experiments. Extracts of leaf discs (0.5 ml) and WFT (0.1 ml – group of 4 WFT removed from an arena) were prepared using a general extraction buffer. The TSWV infection status of groups of WFT from 10–20 arenas of each TSWV inoculation bioassay was determined by ELISA. We measured the optical density (OD) in each ELISA well using a microplate reader (Bio-Rad, model 550) at 405 nm (A_405_). When the A_405_ of an extract was ≥2-fold the average of ELISA-controls (non-infected leaf disc/groups of 4 WFT and buffer blanks), that leaf disc/groups of 4 WFT was determined to be TSWV-infected.

### Statistics

Acylsugar and FA amounts were estimated as least squares means for acylsugar lines or controls using a natural logarithm link function and the generalized linear mixed-effects protocols available in the R packages “lme4” and “emmeans”^47,48^. Individual plants were modeled as random effects nested within lines. Multiple comparisons among tomato and acylsugar lines was done using the “emmeans” package with the default Tukey Honest Significant Difference (Tukey HSD) multiplicity adjustment (α = 0.05).

To illustrate relationships between FA chemical species, a “heatmap”, a color-coded correlation matrix illustrating the magnitude of positive or negative Pearson correlation between FAs, was generated using the R package “ggplot2”^49^. To illustrate the diversity of acylsugar FA chemical profiles among acylsugar lines, we performed agglomerative hierarchical clustering and plotted the dendrograms with the R package “gplots”^50^. Dendrograms for lines were generated by complete linkage of the Euclidean distances between lines with respect to the scaled percentage that each FA chemical species contributed to a line’s overall FA profile. The FA variables were similarly clustered to reflect their distribution across lines.

Oviposition and TSWV inoculation experimental data were analyzed through logistic regression using the R generalized linear model procedure^51^. Eggs were analyzed as binary variables, because, eggs were either oviposited into washed or unwashed leaf. For calculating variance, which may increase as a function of density-dependent choice, eggs from each insect were aggregated into subpopulations. Variance thus represents factors including, but not necessarily limited to: (a) variability between females in strength of response to acylsugars; (b) density-dependent effects on choice (females may prefer a suboptimal substrate if egg accumulation on a preferred substrate is high); and, (c) variability in acylsucrose distribution between leaf discs. Mean differences in estimated preference were calculated as differences in log odds, including confidence intervals based on variance, to detect effects of independent variables. Inoculation of leaf discs with TSWV was analyzed such that experimental replicates were trials, and TSWV infection of leaf discs represented an event. Confidence intervals (95%) were calculated around estimated logits and used to calculate lower and upper limits on a proportion scale. Logistic regression models were fit to the data, and odds ratios were estimated by exponentiating parameter estimates from the logistic function.

Variable selection was conducted to identify the acylsugar FAs that were associated with WFT oviposition and TSWV inoculation. A best-fit multiple logistic regression model was chosen by minimizing AIC. The “brglm2” package was used, maximizing Firth’s penalized likelihood due to quasi-complete separation of data^52^, specifically situations in which no transmission took place for a given combination of experimental treatment levels. Fatty acids whose molar amounts constituted as least 0.5% of at least one line’s overall profile were included in analysis. We did not allow collinear independent variables above r = 0.8 to be present in the models. When multicollinearity existed between two input variables, the one less strongly associated with the response was removed.

## Supplementary information

Supplementary Info

Dataset 1
