# Supplementary material for: A thrips vector of tomato spotted wilt virus responds to tomato acylsugar chemical diversity with reduced oviposition and virus inoculation
Source: Sci Rep. 2019 Nov 20;9:17157. doi: 10.1038/s41598-019-53473-y (PMC6868284; doi:10.1038/s41598-019-53473-y)
Supplement: Supplementary file 1 — Supplementary Info [file 41598_2019_53473_MOESM1_ESM.pdf]

A thrips vector of tomato spotted wilt virus responds to tomato acylsugar chemical diversity with reduced oviposition and virus inoculation

Sulley Ben-Mahmoud[1], Taylor Anderson[2], Thomas M. Chappell[3], John R. Smeda[4], Martha A. Mutschler[2], George G. Kennedy[5], Darlene M. De Jong[2], Diane E. Ullman[1*]

[1] University of California, Department of Entomology and Nematology, Davis, CA 95616, United States of America

[2] Cornell University, Plant Breeding and Genetics Section, School of Integrative Plant Science, Ithaca, NY 14853, United States of America

[3] Texas A&M University, Department of Plant Pathology and Microbiology, College Station, TX 77843, United States of America

[4] University of Florida, Gulf Coast Research and Education Center, Wimauma, FL 33598, United States of America

[5] North Carolina State University, Department of Entomology and Plant Pathology, Raleigh, NC 27695, United States of America

[*]Correspondence to deullman@ucdavis.edu

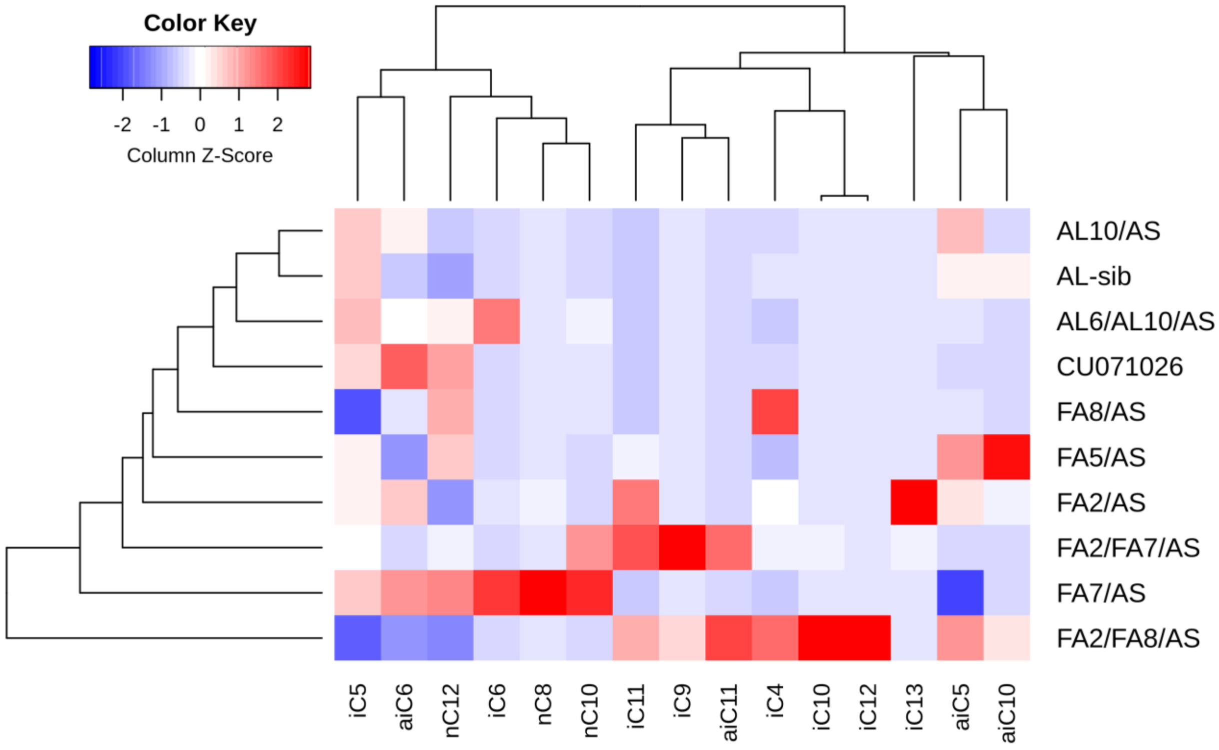

Figure S1. Hierarchical clustering of acylsugar lines by acylsugar fatty acids (FA). Euclidean distance was used to cluster the acylsugar lines by their acylsugar FA profile. Colors are standardized (by Z-scores) across columns, allowing comparisons of the relative proportion of an individual FA across lines, but does not reveal the total amount of acylsugar FA (see Fig. 1B for this information). Comparisons within a row are not relevant, because the colors do not infer total amounts or relative proportion of each FA within a line. Red indicates that the percent accumulation of a FA is high compared to the other lines in the column, while blue indicates that the percent accumulation of a FA is low, or absent, compared to the other lines (see color scale in upper left-hand corner). The length of the dendrogram lines indicates the relative dissimilarity of FA profiles, with longer lines showing greater dissimilarity between clusters than shorter lines. The greater differences are shown among lines with QTL that modify the FAs (Line name prefix = “FA”). While the acylsucrose amount (acylsugar lines with name prefix = “AL”) lines show

little deviation from each other or from their recurrent parent CU071026. The combination of FA QTL in the di-introgression lines FA2/FA7/AS and FA2/FA8/AS show increased diversity of acylsucrose FAs relative to the mono-introgression FA lines.

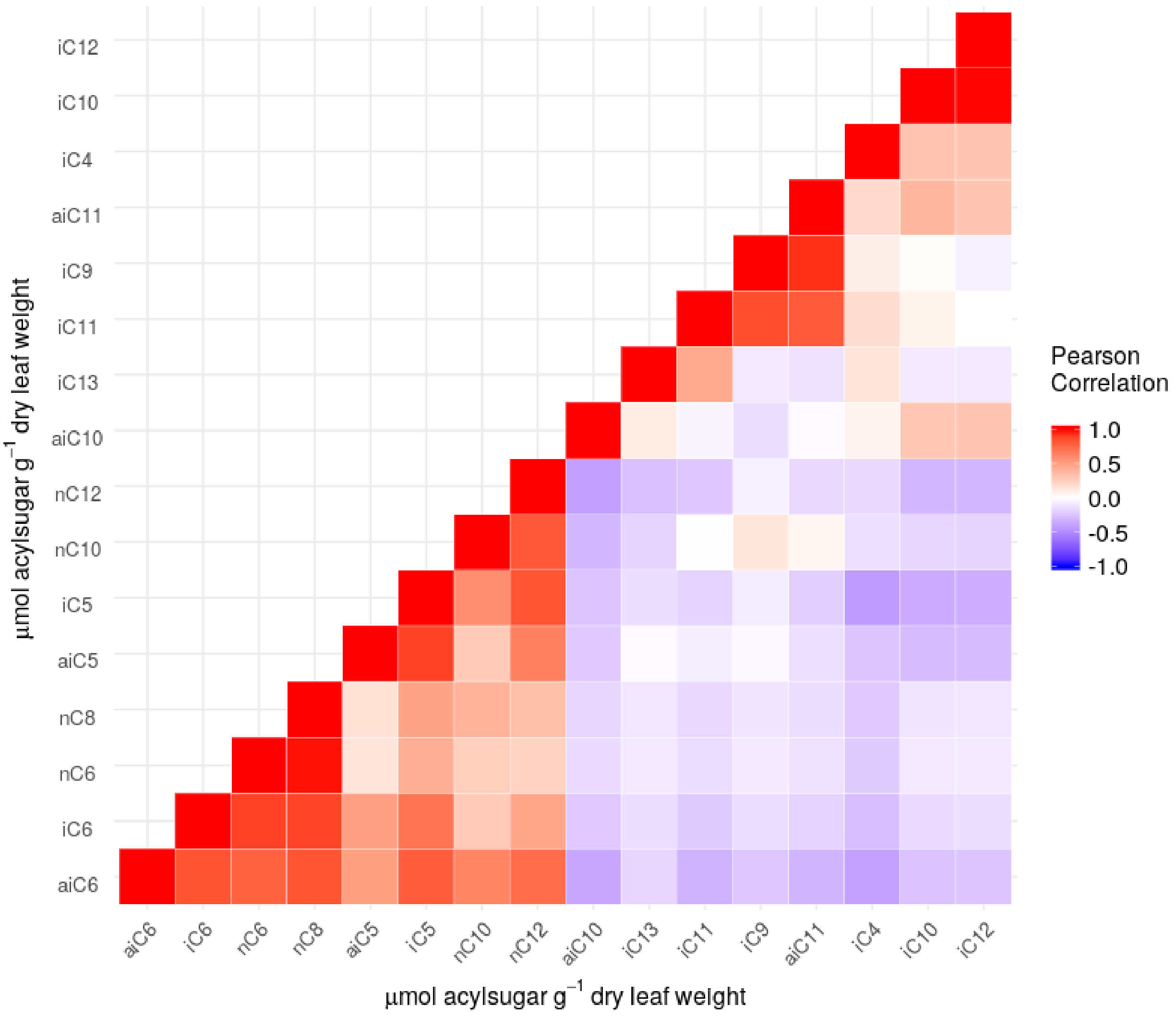

Figure S2. Correlation of acylsugar fatty acid moieties of acylsugar lines. Correlations obtained were generated using the R package "ggplot2". Red indicates a positive correlation and Blue indicates a negative correlation on a scale of 1 to -1.

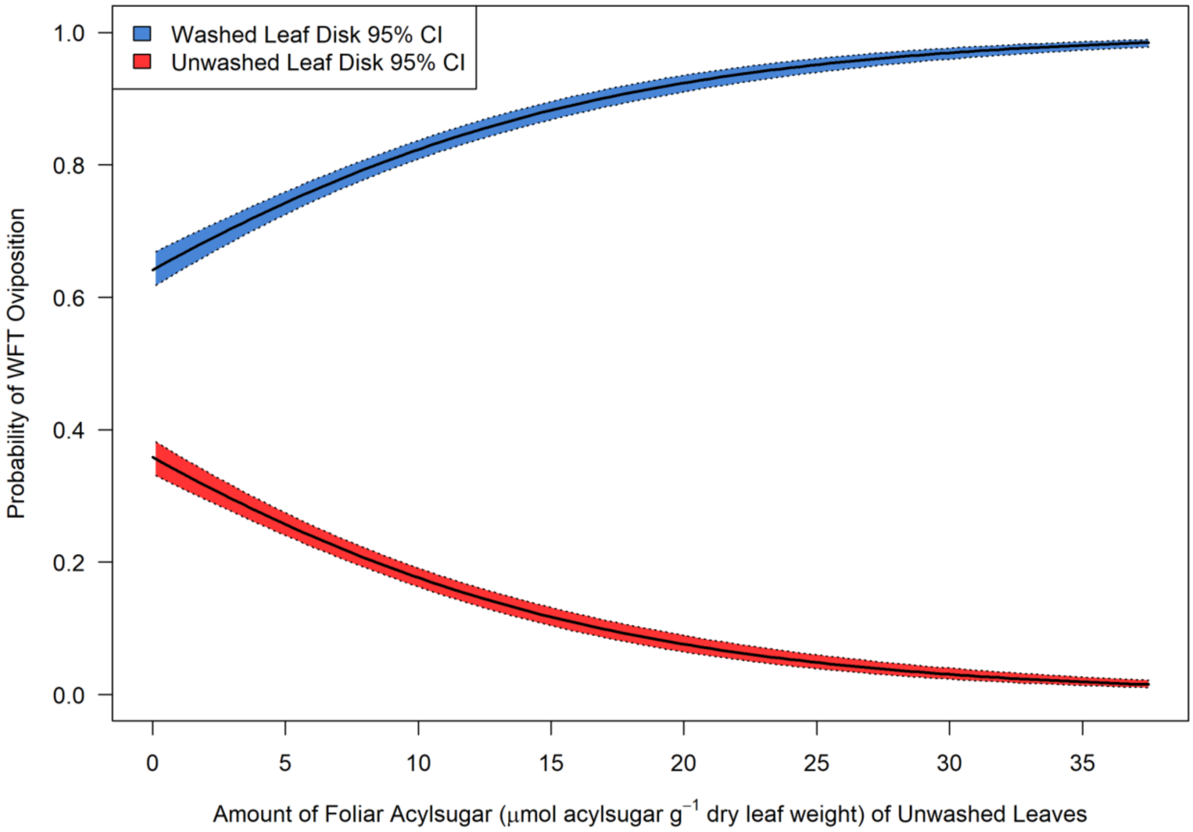

Figure S3. Probability of western flower thrips (WFT) oviposition on unwashed leaf discs decreases with increasing acylsucrose amount. A logistic regression model describes oviposition choice in the aggregated WFT choice experiments as a function of the amount of acylsucrose produced by leaf discs (Null Deviance = 3999.8, Residual Deviance = 3669.0, $P(>|Z|) << 0.001$). Average acylsucrose amounts were obtained from leaves of 12-week-old plants. The amount of acylsucrose corresponds to the expected amount of acylsucrose in the unwashed leaf discs sampled for the washed/unwashed oviposition choice tests. The impact of removal of acylsucrose on probability of oviposition is shown by the difference between washed and unwashed leaf discs across the range of acylsucrose amounts. Blue and red shaded curves

indicate 95% probabilities for WFT oviposition into the washed and unwashed leaf discs. Because an egg can be oviposited onto one of two leaf discs, oviposition was modeled as a binary outcome, yielding probabilities of oviposition onto the washed and unwashed leaf discs that are inversely related. As the amount of foliar acylsucrose increases, deterrence of WFT oviposition is increased, demonstrated by a reduction in the probability of oviposition in the unwashed leaf discs and a corresponding increase in the likelihood of oviposition in the washed leaf discs.

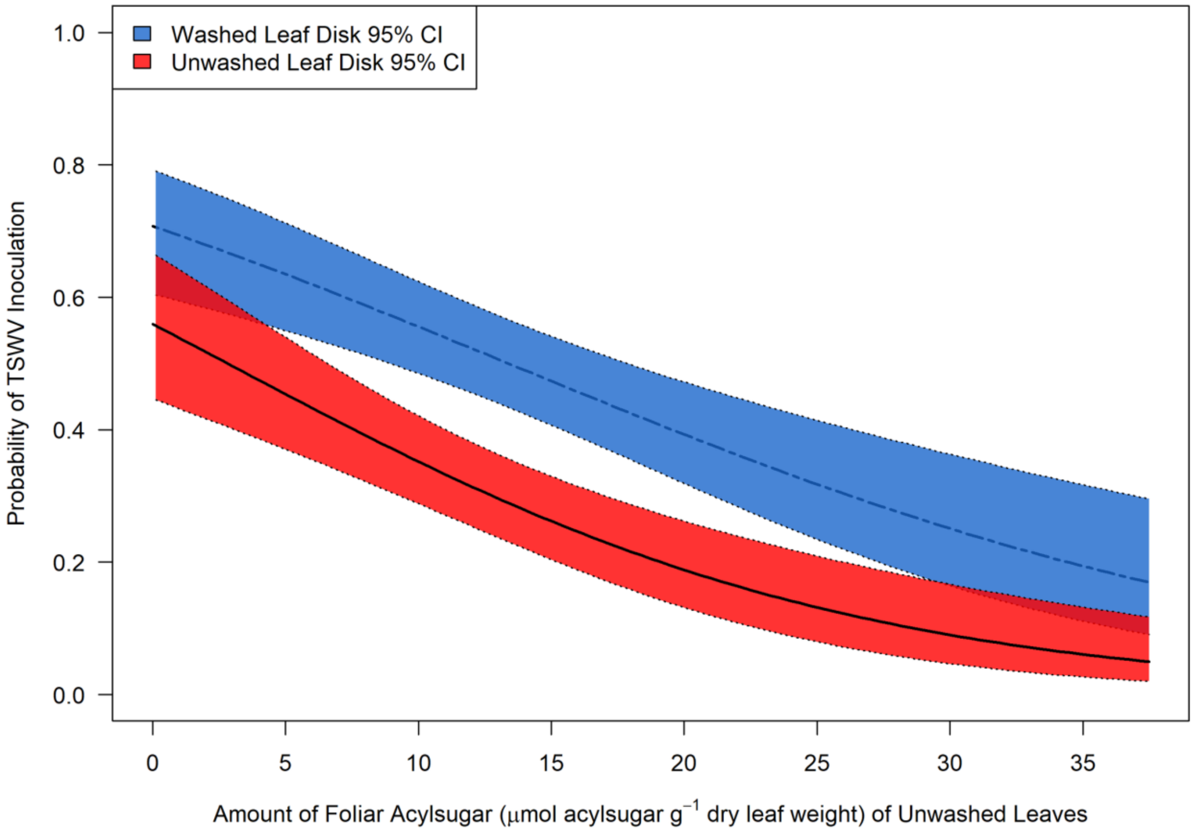

Figure S4. Probability of tomato spotted wilt virus (TSWV) inoculation by western flower thrips (WFT) onto leaf discs decreases with increasing acylsucrose amount. A logistic regression model describes the probability of TSWV inoculation by WFT between washed and unwashed leaf discs in a choice experiment. Average foliar acylsugar amounts were obtained for each line from 12-week-old plants. The amount of foliar acylsucrose corresponds to the expected amount of acylsucrose in the unwashed leaf discs. The probability of TSWV inoculation decreases in both unwashed and washed leaf discs as the average amount of foliar acylsucrose increases (Unwashed curve: Null deviance = 333.18, Residual Deviance = 295.54, P (>|Z|) < 0.001, Washed curve: Null deviance = 386.43, Residual Deviance = 358.87, P(>|Z|) < 0.001).

Blue and red shaded curves indicate the 95% confidence interval for the probabilities that WFT will inoculate TSWV in the unwashed and washed leaf discs, respectively, across a range of acylsucrose amounts. The probability of inoculation into either leaf disc trend similarly, suggesting that the removal of foliar acylsucroses results in a shift in WFT feeding preferences that diminishes as acylsucroses are re-exuded over the course of time.
